# Distribution of corneal spherical aberration in a Tanzanian population

**DOI:** 10.1371/journal.pone.0222297

**Published:** 2019-09-12

**Authors:** Hiroki Asano, Takahiro Hiraoka, Yusuke Seki, Teppei Shibata, Hiromi Osada, Takanori Saruta, Natsuko Hatsusaka, Fukumi Fujikake, Yoshiaki Tabata, Cellina Mhina, Anna Sanyiwa, Tetsuro Oshika, Hiroshi Sasaki

**Affiliations:** 1 Department of Ophthalmology, Tsuchiura Kyodo Hospital Namegata District Medical Center, Ibaraki, Japan; 2 Department of Ophthalmology, Institute of Clinical Medicine, University of Tsukuba, Ibaraki, Japan; 3 Department of Ophthalmology, Kanazawa Medical University, Ishikawa, Japan; 4 Department of Ophthalmology, Nagano Matsushiro General Hospital, Nagano, Japan; 5 Visual Science Course, Department of Rehabilitation, Faculty of Medical Science and Welfare, Tohoku Bunka Gakuen University, Miyagi, Japan; 6 Kagoshima Minami Eye Clinic, Kagoshima, Japan; 7 Department of Ophthalmology, Muhimbili University of Health and Allied Sciences, Dar es Salaam, Tanzania; Keio University School of Medicine, JAPAN

## Abstract

**Purpose:**

To investigate the distribution of corneal spherical aberration (SA) in Tanzanian people of African descent, and to examine the correlation between corneal SA and ocular parameters.

**Design:**

Cross-sectional population-based study.

**Methods:**

Residents aged 40 years and older in three villages in the Mkuranga district in Tanzania were enlisted as study participants. Corneal higher-order aberrations (HOAs) for the right eye were measured with a wavefront analyzer (KR-1W, Topcon) and calculated for the central 6.0-mm zone. Corneal curvature radius (CR), corneal astigmatism, and axial length (AL) were also measured and their correlation with corneal SA was assessed.

**Results:**

The right eyes of 657 participants (336 male, 321 female) were analyzed. The mean age of the subjects was 57.2 ± 10.3 years (mean ± SD). The mean corneal SA (Zernike spherical aberration coefficient C_4_^0^) was 0.188 ± 0.095 μm (-0.242 to 0.613). The SAs in about three-quarters of all subjects were between 0.10 and 0.30 μm. The root mean squares of total corneal HOAs and the third- and fourth-order aberrations were 0.629 ± 0.250 μm, 0.539 ± 0.236 μm, and 0.269 ± 0.110 μm, respectively. Corneal SA showed weak significant correlations with CR (Spearman’s rank correlation coefficient, r = -0.177, p < 0.001), corneal astigmatism (r = -0.142, p < 0.001), AL (r = -0.168, p < 0.001), and age (r = -0.085, p < 0.05).

**Conclusions:**

This finding may be beneficial for selecting aspheric intraocular lens in this population.

## Introduction

In recent times, the goal of cataract surgery has been to not only restore visual acuity but also achieve better quality of vision (QOV), such as improvement in contrast sensitivity. Correcting higher-order aberrations (HOAs) as well as refractive power and astigmatism leads to an optimized retinal image quality [1,2]. Among all the components of HOAs derived from the cornea, only spherical aberration (SA) is correctable with commercially available aspherical intraocular lenses (IOLs). As the value of corneal SA is generally positive [2–5], aspherical IOLs designed to have a negative SA cancel the positive SA of the cornea and thus reduce the whole ocular SA, leading to an improvement in visual function, including contrast sensitivity [6–9]. The degree of compensation of corneal SA using the aspheric IOL varies with the type produced by each company. For example, aspherical IOLs with negative SA, such as Tecnis ZCB00 (Abbott Medical Optics, Santa Ana, CA, USA) and AcrySof IQ SN60WF (Alcon Laboratories Inc., Fort Worth, TX, USA) are designed to compensate for positive corneal SA by -0.27 and -0.20 μm, respectively.

Holladay et al. [2] reported that the mean value of corneal SA was 0.27 μm in Caucasian individuals with cataract. Several researchers also reported the distribution of corneal SA during middle and advanced ages in Caucasian (0.27–0.33 μm) [2,3,10–12], Asian (0.20–0.31 μm) [4,5,13], and Middle-Eastern (0.28–0.32 μm) [14,15] individuals, indicating ethnic variations. However, to our knowledge, no study directly comparing the differences in corneal SA among different races in middle-aged and elderly people has been conducted. Besides, the distribution of corneal SA in sub-Saharan African people has not yet been reported.

Tanzania, which is located in east Africa, is one of the least developed countries in the world. In the past 25 years, the population has doubled to more than 50 million, and the average life expectancy has increased [16]. With the increase in the elderly population in the future, an increase in the occurrence of cataracts and the number of cataract operations can be expected. In Tanzania, approximately 70% of the people live in rural areas. Ideally, aspherical IOLs should be personalized for each patient for cataract surgery by measuring the corneal SA [1,17,18], but it is difficult for aberration measurement equipment to be available at each facility even in developed countries, and more so in African countries. Therefore, understanding the distribution of corneal aberrations is useful for selecting the type of the IOL to be inserted. In this study, we aimed to investigate the distribution of corneal SA in a Tanzanian population in order to assess whether the amount of negative SA in the currently available aspherical IOLs is suitable for this population. In addition, we examined the correlation between corneal SA and common preoperative ocular parameters such as corneal curvature radius (CR), corneal astigmatism, and axial length (AL).

## Materials and methods

### Study population

This study was performed as part of a cross-sectional population-based study conducted from August to September in 2014 in the Mkuranga district hospital in the Pwani region. The Mkuranga district is a rural area where approximately 85% of the people are engaged in agriculture. Out of the 18 wards of the Mkuranga district, 3 wards were selected, and one typical village within a driving distance of 1 hour by car from the district hospital was further chosen from each ward. A total of 1,276 adult residents, aged 40 years and over, in three villages (Tengelea, Kiziko, and Msfini-Kidete) were enlisted as study participants based on the records provided by village health workers.

### Data collection

All ocular biometry measurements, including HOAs, were performed by ophthalmologists and orthoptists. CR, corneal astigmatism, and refraction were measured using an auto kerato-refractometer (KR-8900; Topcon Co., Tokyo, Japan). Uncorrected visual acuity (UCVA) and best-corrected visual acuity (BCVA) were assessed with a System Chart SC-1600 (NIDEK Co., Ltd, Aichi, Japan). After dilation with a combination of tropicamide and phenylephrine, ACD and AL were measured using a non-contact optical biometry device (AL-Scan, NIDEK). This device provides highly repeatable and reproducible estimates of ACD and AL that are in good agreement with the measurements obtained with IOLMaster (Carl Zeiss Meditec, Jena, Germany) [19]. Corneal and ocular HOAs including SA (Zernike spherical aberration coefficient C_4_^0^) were measured three times for the right eye with a Hartmann-Shack wavefront analyzer with Placido disk topographer (KR-1W, Topcon Co.) and were calculated for the central 6.0-mm zone. Anterior segment and fundus examinations were performed using a slit-lamp (SL-D8Z, Topcon Co.) and indirect ophthalmoscope to check ocular health.

### Data analysis

We excluded cases with BCVA worse than 0.1 (20/200), corneal opacity, ocular phthisis, moderate to advanced pterygium, less dilatation, poor fixation, and history of ocular surgery or trauma. For wavefront aberration data, three measurements were averaged in each eye.

### Statistical analysis

All statistical analyses were performed using IBM SPSS statistics version 25 (IBM Japan, Tokyo, Japan). Correlations between corneal C_4_^0^ and each parameter such as CR, corneal astigmatism, and AL were assessed using the Spearman’s rank correlation analysis. P-value < 0.05 was considered to indicate statistical significance.

### Ethics

Written informed consent was obtained from each participant. This study was conducted according to the Declaration of Helsinki and was approved by the ethics committees of Muhimbili University of Health and Allied Sciences (approval number: MU/DRP/AEC/Vol.XVIII/136), Kanazawa Medical University (2014–206), and University of Tsukuba (2015–989).

## Results

**Fig 1:**
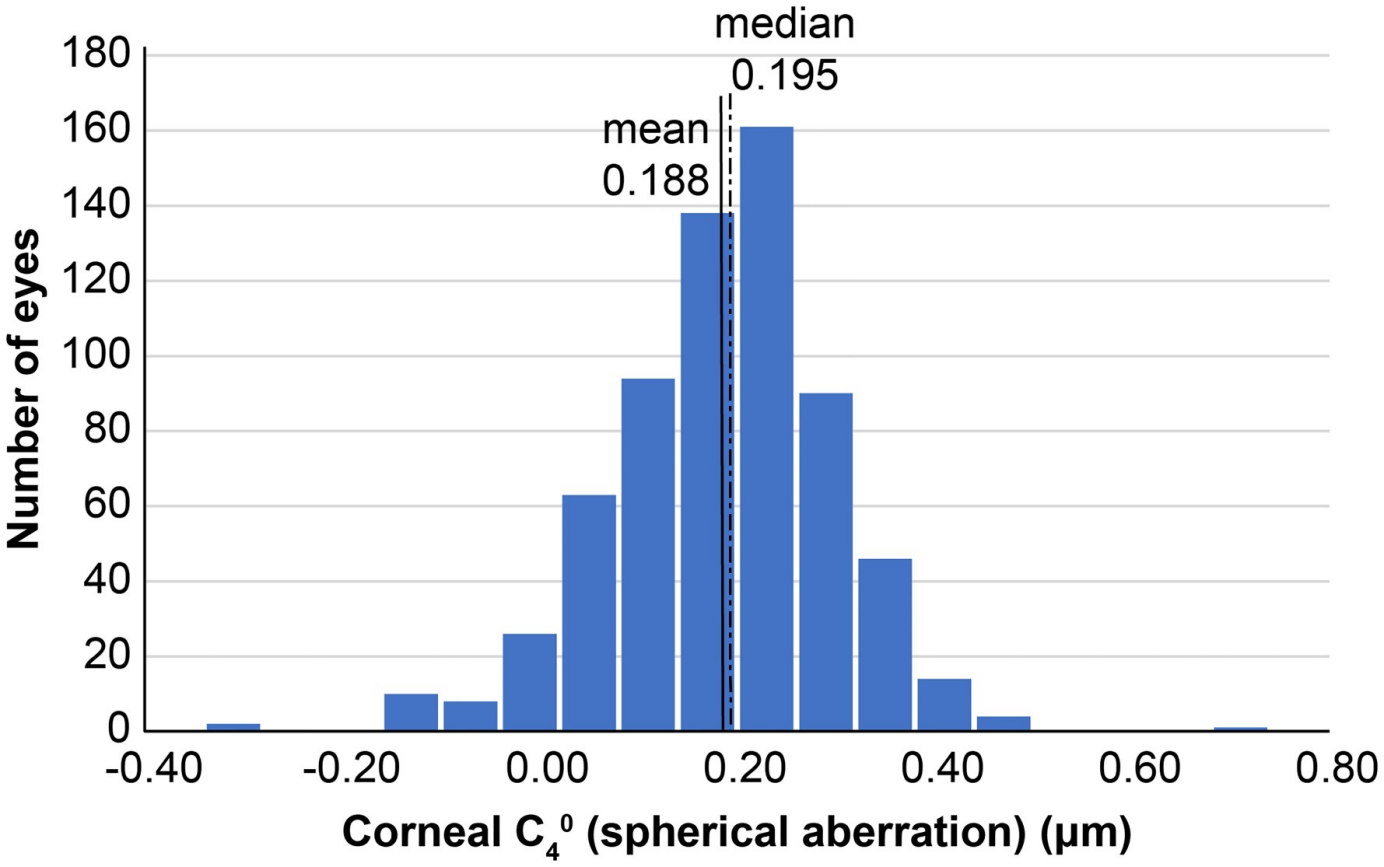
Distribution of corneal spherical aberration (SA; C_4_^0^). The mean corneal SA was 0.188 ± 0.095 μm, and is represented by the thick solid line. The median value was 0.195 μm, and is represented by the dotted line.

A total of 937 individuals participated in the entire survey. The participation rate was 73.4% and all participants were of African descent. After applying exclusion criteria, 657 right eyes (336 male, 321 female) were included in the analysis. The age of the subjects was 57.2 ± 10.3 (mean ± standard deviation) years. CR was 7.67 ± 0.29 mm; corneal astigmatism was 0.90 ± 0.60 diopters; and ACD and AL were 3.07 ± 0.33 mm and 23.03 ± 0.84 mm, respectively (Table 1). The mean corneal SA was 0.188 ± 0.095 μm (range -0.242 to 0.613), and the median value was 0.195 μm (Fig 1). When SA was divided at every 0.05 μm, the mode value was between 0.20–0.25 μm. The distribution of corneal SA did not exhibit a normal Gaussian curve (Kolmogorov-Smirnov normality test, p = 0.001). The root mean square (RMS) of the corneal total HOAs and the third- and fourth-order aberrations were 0.629 ± 0.250 μm (range 0.204 to 1.896), 0.539 ± 0.236 μm (0.123 to 1.774), and 0.269 ± 0.110 μm (0.052 to 0.920), respectively.

**Table 1:** Demographic and clinical characteristics of subjects (n = 657).

	Mean ± SD	Min	Max
Age	57.2 ± 10.3	40	91
Sex (male: female)	336:321	-	-
UCVA (logMAR)	0.404 ± 0.428	-0.176	2.000
BCVA (logMAR)	0.160 ± 0.281	-0.176	1.000
Corneal curvature radius (mm)	7.67 ± 0.29	6.93	8.69
Corneal astigmatism (diopter)	0.90 ± 0.60	0.00	4.00
Axial length (mm)	23.03 ± 0.84	20.33	27.00
Anterior chamber depth (mm)	3.07 ± 0.33	1.99	3.94
Corneal S3 (μm)	0.539 ± 0.236	0.123	1.774
Corneal S4 (μm)	0.269 ± 0.110	0.052	0.920
Corneal C_4_^0^ (Spherical aberration) (μm)	0.188 ± 0.095	-0.236	0.613
Corneal total HOA (μm)	0.629 ± 0.250	0.204	1.896

UCVA = uncorrected visual acuity, BCVA = best-corrected visual acuity, S3 = root-mean-square (RMS) of third-order aberrations, S4 = RMS of fourth-order aberrations, C_4_^0^ = spherical aberrations, HOA = higher-order aberrations, SD = standard deviation

**Fig 2:**
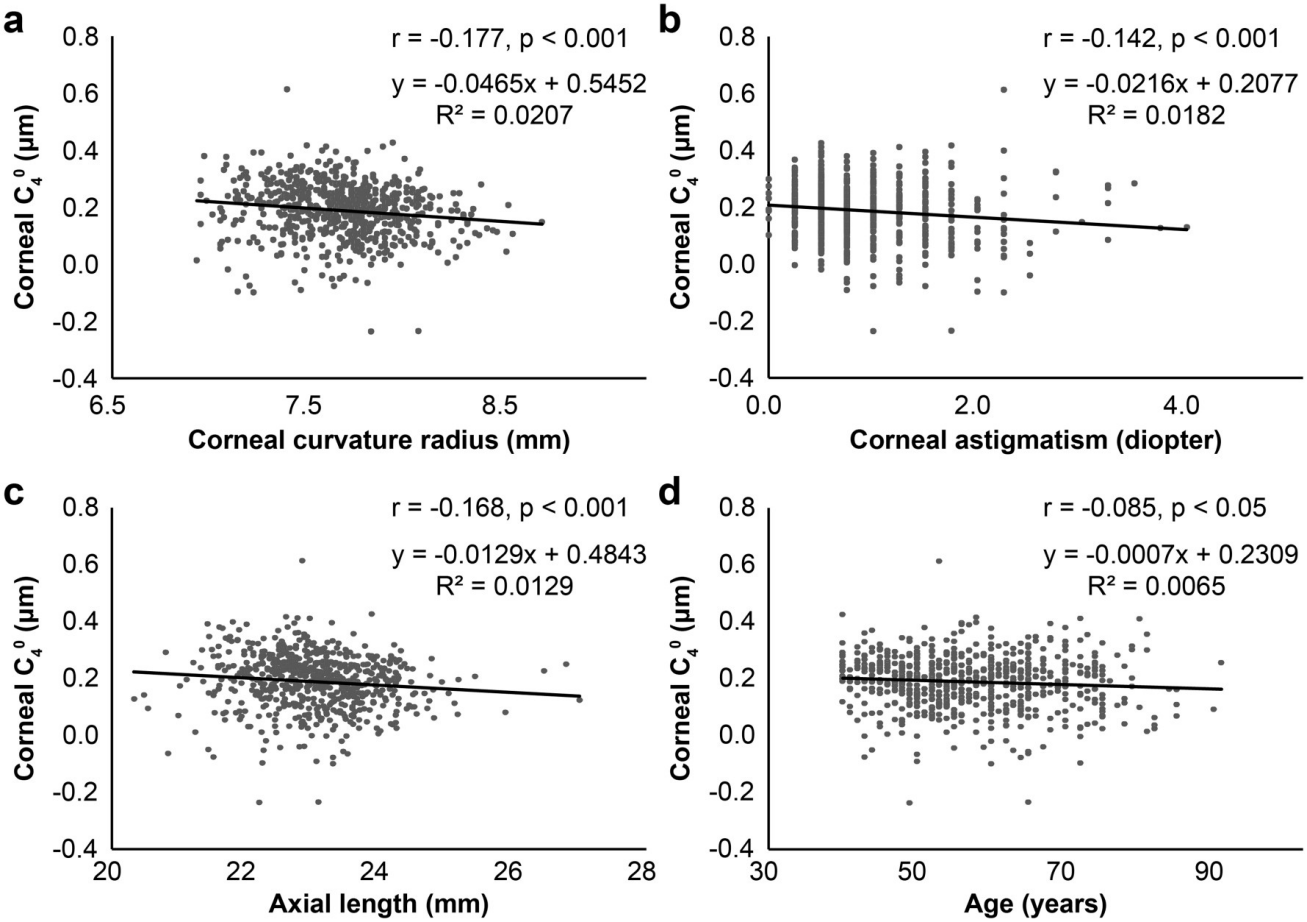
Relationships between corneal spherical aberration (C_4_^0^) and ocular parameters, and age. (a) Scatterplots demonstrating corneal curvature radius and C_4_^0^. A significant correlation was observed between the two (Spearman’s correlation coefficient, r = -0.177, p < 0.001). (b) Scatterplots demonstrating corneal astigmatism and C_4_^0^. A significant correlation was observed between the two (r = -0.142, p < 0.001). (c) Scatterplots demonstrating axial length and C_4_^0^. A significant correlation was observed between the two (r = -0.168, p < 0.001) (d) Scatterplots demonstrating age and C_4_^0^. A significant correlation was observed between the two (r = -0.085, p < 0.05).

In correlation analyses, corneal SA showed significant correlations with CR (Spearman’s correlation coefficient, r = -0.177, p < 0.001), corneal astigmatism (r = -0.142, p < 0.001), and AL (r = -0.168, p < 0.001). A weak correlation was also found between SA and age (r = -0.085, p < 0.05) (Fig 2).

## Discussion

We conducted a population-based study in Tanzania and, to the best of our knowledge, reported the distribution of corneal SA in an African population in the sub-Saharan area for the first time. The mean corneal SA was 0.188 ± 0.095 μm at the mean age of 57.2 ± 10.3 years. The corneal SAs in 38.4% of eyes (251 / 657) were between 0.20 and 0.30 μm and those in 35.3% (232 eyes) were between 0.10 and 0.20 μm; therefore, the SAs of about three-quarters of all the subjects were between 0.10 and 0.30 μm. When the SAs were divided at every 0.05 μm, both the mean and median values were between 0.15 and 0.20 μm, and the mode value was between 0.20 and 0.25 μm. The SAs in 3.0% (20 eyes) were negative values, which could be attributed to the smaller mean and median values compared to the mode value.

**Fig 3:**
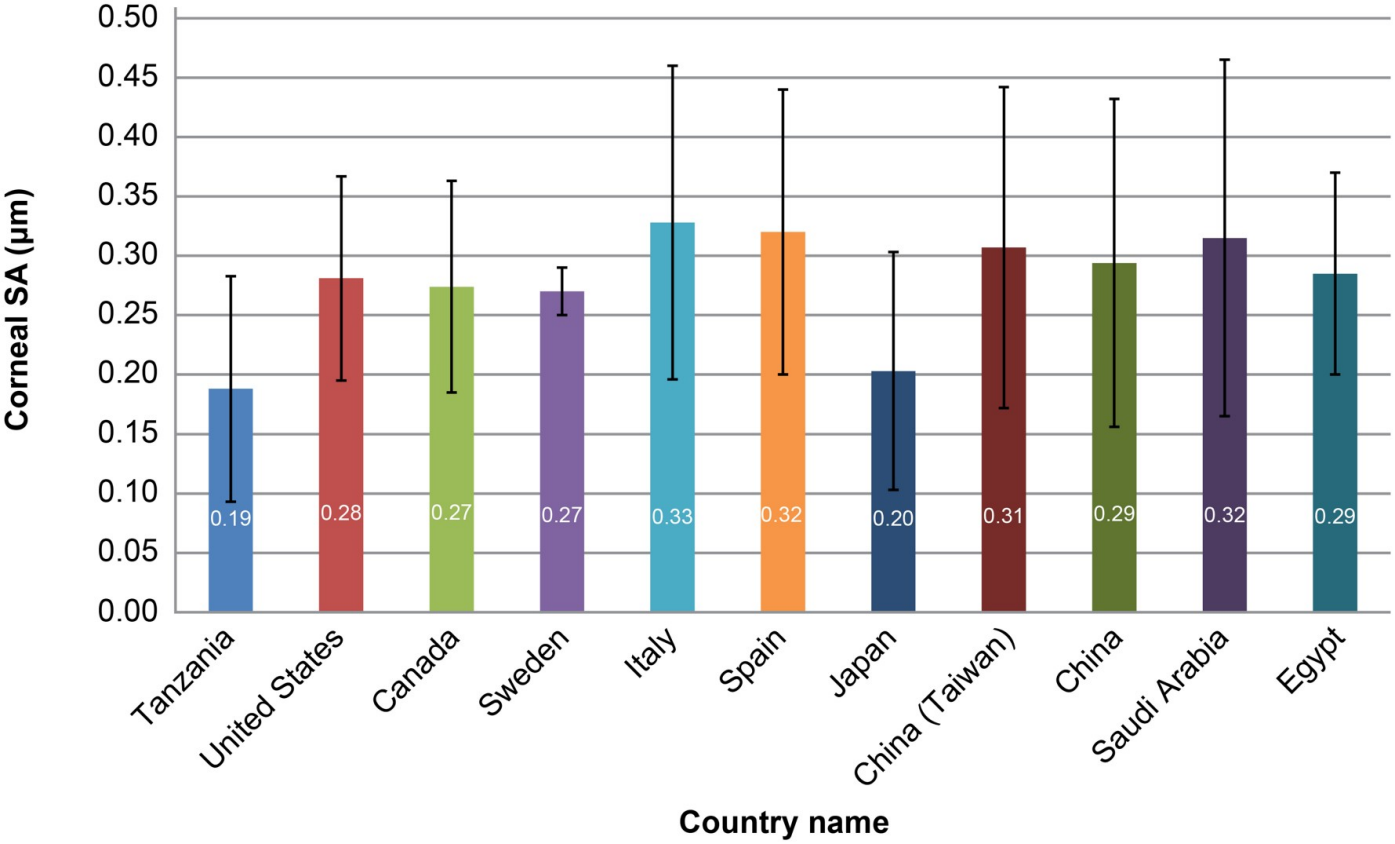
Comparison of corneal spherical aberrations (SA) at the 6-mm optical zone between this study and previous reports in individuals over the mean age of 40 years [2–5,10–15]. The mean value of corneal SA in Tanzanian individuals was 0.19 μm, which tended to be small among the previously reported values.

Fig 3 shows the comparisons of corneal SAs in the central 6-mm zone between the current study and previous studies in subjects over a mean age of 40 years, and each study analyzing over 50 eyes. The mean value of 0.19 μm in Tanzanian individuals tended to be small compared with the value of 0.27–0.33 μm in Caucasian [2,3,10–12], Middle-Eastern [14,15], and most Asian individuals [5,13] (S1 Table).

As for the dispersion of corneal SA, the standard deviation was 0.10 μm in this study, and the minimum and maximum were -0.242 and 0.613 μm, respectively, showing a big difference between individuals. As shown in Fig 3, the standard deviations of SA were reported to be more than 0.08 μm, except in one study. This indicates that considerable variation of corneal SA is observed in Tanzanian individuals as well as in other ethnicities.

In this research, KR-1W (Topcon) was used for the measurement of HOAs. Since the devices used for measuring HOAs were different among the studies shown in S1 Table, the reported aberration values could not be compared directly. With regard to the reliability of KR-1W in comparison with other equipment, Hao et al. [20] reported that the corneal HOAs measured by the KR-1W and iTrace showed no statistically significant differences (p > 0.05). In addition, López-Miguel et al. [21] and Xu et al. [22] reported that the KR-1W device provides excellent repeatability and intersession reproducibility in the measurement of corneal SA. Hence, our results seemed sufficiently accurate and reliable, and it is unlikely that corneal SA of Tanzanian individuals was underestimated in this study. For reference, the mean value of the total corneal HOAs was 0.63 ± 0.25 μm in this study, which is similar to the value of 0.65 ± 0.46 μm in a previous study by Guirao et al. [12] that used videokeratoscopy (Eyemap EH-290; Alcon, Fort Worth, TX). However, the mean value of corneal SA was 0.19 ± 0.10 μm in this study, which is much smaller than the value of 0.32 ± 0.12 μm in the study by Guirao et al. [12]. Thus, the corneal SAs seemed to be relatively small in the Tanzanian individuals in this study.

We examined the correlations between ocular parameters and corneal SA. A weak negative correlation was found between CR and corneal SA (r = -0.177, p < 0.001) in this study, which was compatible with that in a previous study [3]. Although corneal astigmatism showed a significant correlation with corneal SA in this study (r = -0.142, p < 0.001), Zhao et al. [22] reported that corneal SA of astigmatic corneas was similar to those of nonastigmatic corneas at a mean age of 42.6 ± 11 years. AL also showed a weak negative correlation with corneal SA (r = -0.168, p < 0.001) in our study, similar to a previous report [4]. In this study the correlation of corneal SA with age was very weak (r = -0.085, p < 0.05). The correlation between corneal SA and age remains somewhat controversial. Some researchers reported that there was no correlation [23,24] or poor correlation [3]; however, in a study conducted in China by Kemraz et al. [25] it was reported that corneal SA increased non-linearly with age and became more positive after 39 years of age. The correlations may be different among different ethnicities.

The limitation in this study was a low participation rate for a population-based study (participation rate of only 73.8% [937 participants]). In addition, the subjects available for current data analysis with regard to HOAs decreased to 657 eyes, owing to the exclusion criteria. However, compared with other previous studies on corneal SA, the population in this study was large, and the obtained results can be considered to be meaningful.

In summary, this study was the first to document the corneal SA in sub-Saharan African individuals. The mean value of the corneal SA in a large number of Tanzanian individuals tended to be small among the previously reported values. We observed that corneal SA varied widely between different subjects. If an aspherical IOL with a SA of -0.27 μm such as Tecnis ZCB00 is selected, the ocular SA may show overcorrection. IOLs with milder asphericity may be suitable for Tanzanian individuals.

## Supporting information

S1 Dataset Detailed information for all subjects. (XLSX)

S1 Table Comparison of corneal spherical aberrations between this study and previous reports. (DOCX)
