# Supplementary material for: Distribution of corneal spherical aberration in a Tanzanian population
Source: PLoS One. 2019 Sep 12;14(9):e0222297. doi: 10.1371/journal.pone.0222297 (PMC6742233; doi:10.1371/journal.pone.0222297)
Supplement: S1 Table — (DOCX) [file pone.0222297.s002.docx]

**S1 Table. Comparison of corneal** **spherical aberrations between this study and previous reports.**

| Study^*^ | Location | n | Age (years)  Mean ± SD (Range) | Corneal HOA (RMS)  Mean ± SD (μm) | Corneal SA  Mean ± SD (μm) | Corneal SA  Range (μm) |
| --- | --- | --- | --- | --- | --- | --- |
| Sub-Saharan |  |  |  |  |  |  |
| Current study | Tanzania | 657 | 57.2 ± 10.3 | 0.629 ± 0.250 | 0.188 ± 0.095 | -0.242 to 0.613 |
| Caucasian |  |  |  |  |  |  |
| Wang^10^ | United States | 228 | 50 ± 17 | 0.479 ± 0.124 | 0.281 ± 0.086 | 0.055 to 0.544 |
| Beiko^3^ | Canada | 602 | 65.6 ± 15.9 |  | 0.274 ± 0.089 | 0.041 to 0.632 |
| Holladay^2^ | Sweden | 71 | 74 (35 to 94) |  | 0.27 ± 0.02 | < -0.10 to > 0.75 |
| de Sanctis^11^ | Italy | 149 | 71.7 ± 9.1 |  | 0.328 ± 0.132 |  |
| Guirao^12^ | Spain | 70 | 70 ± 12 | 0.65 ± 0.46 | 0.32 ± 0.12 |  |
| East Asian |  |  |  |  |  |  |
| Shimozono^4^ | Japan | 257 | 74.3 ± 8.7 |  | 0.203 ± 0.100 | -0.103 to 0.497 |
| Lai^5^ | China (Taiwan) | 413 | 66.8 ± 10.6 |  | 0.307 ± 0.135 | -0.200 to 0.840 |
| Li^13^ | China | 155 | (50 to 89) |  | 0.294 ± 0.138 |  |
| Middle-Eastern |  |  |  |  |  |  |
| Al-Sayyari^14^ | Saudi Arabia | 100 | (50 to 85) |  | 0.315 ± 0.150 | 0.017 to 0.708 |
| Assaf^15^ | Egypt | 56 | 66.1 ± 9.2 |  | 0.28 ± 0.08  0.29 ± 0.09  (2 groups) |  |

HOA = higher-order aberration, RMS = root mean square, SA = spherical aberration

*first author of study
